# Two Rare Complications in One Patient: Acquired von Willebrand Syndrome Associated with Intracranial Plasmacytoma

**DOI:** 10.1155/2019/7609308

**Published:** 2019-08-27

**Authors:** H. Auge, C. Yguel, E. Schmitt, B. Frotscher, H. Busby-Venner, R. Morizot, C. Moulin, P. Feugier, A. Perrot, L. Filliatre-Clement

**Affiliations:** ^1^Hematology Department, University Hospital of Nancy, 5 Rue du Morvan, 54500 Vandoeuvre les Nancy, France; ^2^Histopathology Department, University Hospital of Nancy, 5 Rue du Morvan, 54500 Vandoeuvre les Nancy, France; ^3^Neuroradiology Department, University Hospital of Nancy, 29 Avenue du Maréchal de Lattre de Tassigny, 54035 Nancy, France; ^4^Hemostasis Department, University Hospital of Nancy, 5 Rue du Morvan, 54500 Vandoeuvre les Nancy, France

## Abstract

Here, we describe a rare case of acquired von Willebrand syndrome (VWS) associated with intracranial plasmacytoma. The literature includes reports of a few cases of plasmacytoma with central nervous involvement, but none of them with acquired VWS. Diagnosis was made based on a stereotaxic intracerebral biopsy. During this biopsy, a ventriculoperitoneal shunt was established, which was complicated with abnormal bleeding. Subsequent hemostasis assessment related to hemopathy revealed acquired von Willebrand disease. The patient received induction therapy with bortezomib, thalidomide, and dexamethasone (VTD), followed by high-dose melphalan chemotherapy and autologous stem cell transplantation, and then VTD consolidation, and finally maintenance with lenalidomide. Our patient currently remains in very good partial response without neurological symptoms after 4 months of maintenance. The patient is free of progression 14 months after their original presentation.

## 1. Introduction

Multiple myeloma (MM) is a rare hematological malignant pathology, but its incidence has increased over recent years (5-6 cases per 100,000 population). Therapeutic advances have increased the 5-year survival rate to around 70% [1]. Patients rarely present with central nervous system (dural or extradural) involvement at diagnosis, with only a few such cases described in the literature [2]. MM can also be complicated with an acquired von Willebrand syndrome (VWS). Although von Willebrand disease is the most common hereditary hemorrhagic pathology (affecting up to 1% of the general population), the acquired form of VWS is an unusual complication of certain lymphoproliferative disorders, particularly MM [3].

## 2. Case Presentation

**Figure 1:**
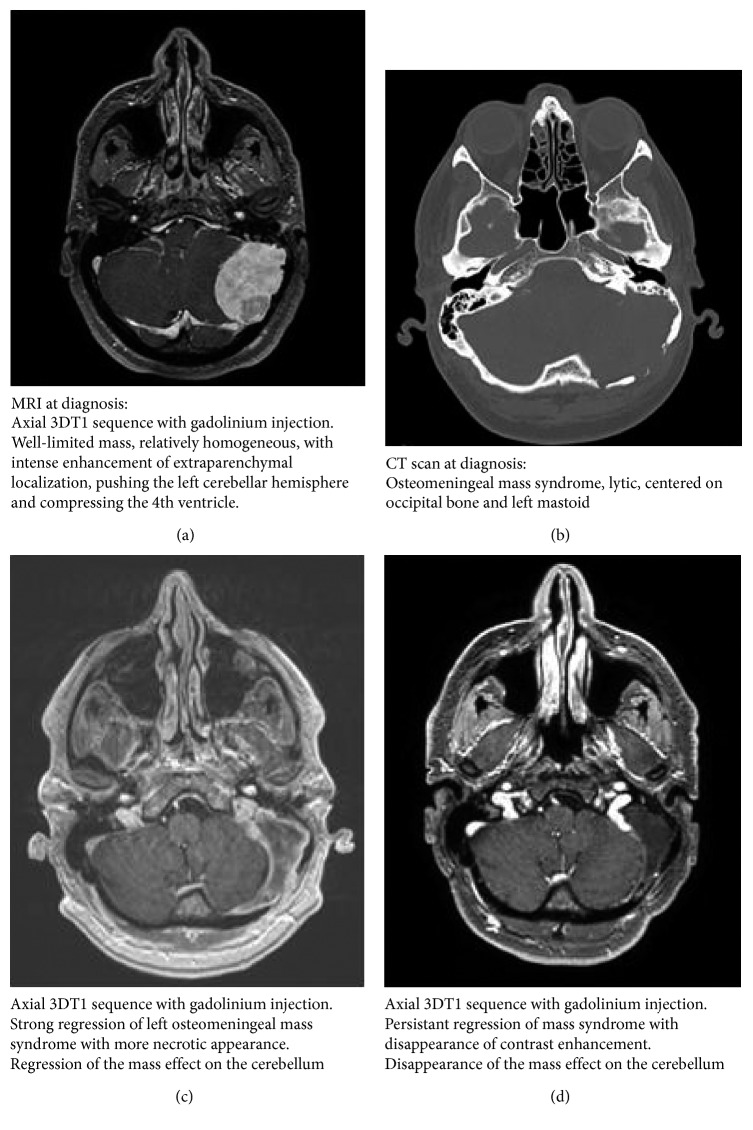
(a) MRI at diagnosis. (b) CT scan at diagnosis. (c) Follow-up MRI after two courses of bortezomib, thalidomide, and dexamethasone. (d) Follow-up MRI after two courses of lenalidomide maintenance.

Here, we report the case of a 48-year-old Caucasian male with no previous relevant medical/surgical history, who was admitted to neurology services due to intermittent headaches, dysgeusia, and persistent dizziness for two months. His symptoms also included spontaneously resolving gum bleeding. An MRI was performed (Figure 1(a)), revealing a voluminous left extraparenchymal posterior fossa tumor, complicated by occipital osteolysis and hydrocephalus, without distant metastatic lesions. Computerized tomography (CT) scanning revealed no other suspicious lesion (Figure 1(b)).

A stereotaxic intracerebral biopsy was performed, and at the same time, a ventriculoperitoneal shunt was established to control intracranial hypertension. These procedures were complicated by a voluminous right parietal hematoma with mass effect and falcorial and temporal commitment, resulting in a secondary epileptic seizure. No hemostasis report was available before this intervention.

To explore this major bleeding, a hemostasis assessment was performed. This revealed a prolonged activated partial thromboplastin time (APTT) of 44 s (normal value (NV): 23–35 s) and a decreased factor VIII level (FVIII: 10%; NV: 60–150%). In addition, the level of von Willebrand factor antigen was low (vWF : Ag < 10%; NV > 60%), and the activity of vWF ristocetin cofactor was severely decreased (vWF : RCo < 4%, undetectable; NV > 60%), indicating an acquired von Willebrand syndrome (VWS).

Biological evaluation, including serum protein electrophoresis, revealed a monoclonal gammopathy IgG kappa of 16 g/L, associated with high kappa-free light chain of 109 mg/L (NV, 3.3–19.4 mg/L). The lambda-free light chain level was 37.2 mg/L (NV, 5.7–26.3 mg/L), and the kappa/lamba ratio was 2.94 (NV, 0.26–1. 65). Complete blood count (CBC), renal function, and corrected serum calcium were normal. We detected a Bence-Jones proteinuria, with global proteinuria of 1.73 g/L, and B2 microglobulin of 1.88 mg/L (NV, 0.9–2 mg/L). The albumin level was 12.3 g/L (NV, 35–52 mg/L), and the LDH level was 265 U/L (NV, <248 U/L).

**Figure 2:**
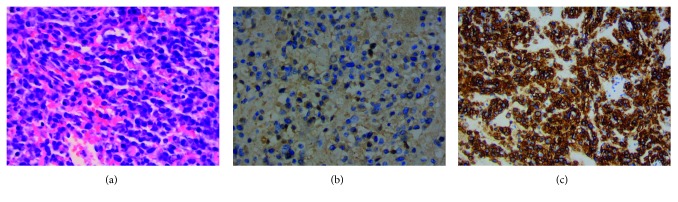
(a) Neoplastic plasma cell proliferation. Hematoxylin-eosin-saffron (HES) coloration, ×400 magnification. (b) Monotypic plasma cell proliferation. Anti-kappa antibody, ×400 magnification. (c) CD138 expression by plasma cells. Anti-CD138 antibody, ×400 magnification.

Histological examination of the biopsy supported a diagnosis of plasmacytoma (Figures 2(a)–2(c)), revealing the following atypical plasma cells proliferation markers: CD38^+^, MUM–IRF4^+^, positive expression of kappa-free light chain (KP-53 clone) without expression of lambda-free light chain (K22-Y clone), CD20^−^, CD3^−^, and proliferation index ki67 of 5%. Bone marrow aspiration showed 5% atypical plasma cells. Serum and bone marrow immunophenotyping were not performed. Cytogenetic analysis supported the conclusion of a standard risk, without *t*(4;14) or del(17p). The revised prognostic score R-ISS was evaluated to be 2.

Prior to myeloma confirmation, the initial case management comprised symptomatic treatment, including evacuation of intraparenchymal hematoma, and establishment of an external ventricular bypass. Immediately before this surgical intervention, FVIII was administered in association with VWF (KOVALTRY® 70 UI/kg + WILFACTIN® 52 UI/kg UI). Immediately after surgical intervention, FVIII (KOVALTRY® 70 UI/kg) was administered without VWF. During the night, this treatment was switched to recombinant factor VII (NOVOSEVEN® 86 *μ*g/kg). FVIII treatment increased the circulating FVIII level to 59% with persisting low levels of vWF : Ag (11%) and vWF : Rco (4%). Administration of high-dose intravenous immunoglobulins (CLAYRIG® 0.8 mg/kg) allowed the FVIII level to increase to >200%, with a vWF : Ag level of 68% and vWF : Rco level of 70%, causing the bleeding to stop. The patient's FVIII and VWF levels remained normal for 3 weeks. At 3 weeks after the first infusion, VWF activity decreased to 55%, with a vWF : Ag level of 120% and normal FVIII at 135%, without bleeding event, necessitating another treatment with intravenous immunoglobulins before a new surgical intervention (laparoscopy) was required to eliminate peritonitis by infection of the external ventricular bypass valve. Subsequently, we observed normalization of hemostasis (FVIII > 150%; VWF activity > 60%; vWF : RCo > 60%).

For symptomatic myeloma with extraparenchymal plasmacytoma developed at the expense of the occipital bone, the specific treatment started with 4 days of administration of 40 mg of dexamethasone treatment alone. This was followed by the induction of four 28-day cycles of VTD plus bortezomib 1.3 mg/m^2^ on D1, D4, D8, and D11; thalidomide 100 mg continuously; and dexamethasone 40 mg on D1-D2, D8-D9, D15-D16, and D22-D23. After one cycle of VTD, the ventriculoperitoneal shunt could be removed. After two cycles, MRI showed regression of the plasmacytoma, which was considered complete (Figure 1(b)). After the third cycle, we observed a very good partial response, with nonmeasurable serum and urine M-proteins and normalization of the light chain ratio, as well as complete normalization of the hemostasis balance parameters.

The patient then received high-dose melphalan chemotherapy and autologous stem cell transplantation, followed by two VTD consolidation courses, and then maintenance with lenalidomide. After two courses of maintenance, MRI revealed a net decrease of the extraparenchymal posterior fossa tumor, with a greater necrotic aspect and less enhancement (Figure 1(c)). To date, our patient remains in VGPR without neurological symptoms after 4 months of maintenance. The patient is free of progression 14 months after their original presentation.

## 3. Discussion

To our knowledge, this is the first reported case of extraparenchymal intracranial plasmacytoma associated with acquired VWS. Kumar et al. [4] suggested that physiopathological mechanisms of acquired VWS are poorly understood, but its occurrence with malignant hematologic disorders, especially dysproteinemias, has been described. [5–11]. Acquired VWS is usually similar to the “2A” type inherited disease, in that it involves decreases of both the activity and concentration of vWF (vWF : RCo and vWF : Ag) [12]. In addition, type 1 or type 3 VWD can also be detected in individuals in whom acquired VWS is associated with abnormal synthesis of vWF (e.g., hypothyroidism) [12], and optimally, a multimeric analysis completes the diagnosis of acquired VWS. Acquired VWD in MM is based on several mechanisms: antibodies specifically directed against functional domains of VWF [11, 13], the monoclonal protein can also accelerate immunologic clearance of VWF [14], adsorption of VWF by malignant cells [15], and finally a GpIb-mediated selective adsorption of VWF on malignant cells [16].

Intravenous immunoglobulin (IVIg) has shown efficacy for treatment of acquired VWS with type immunoglobulin (Ig) G monoclonal gammopathies of undetermined significance (MGUS) [17, 18]. Such treatment can reportedly induce a prompt and sustained increase of FVIII/VWF activities and shorten the bleeding time for at least 15–20 days for all IgG-MGUS with a correction of VWF multimeric structure [6]. Data from the International Society on Thrombosis and Hemostasis (ISTH) registry indicate that patients exhibiting acquired VWS along with lymphoproliferative disease (48% of patients with acquired VWS) may also benefit from IVIg therapy [6, 18]. Normalization of plasma vWF activity is usually observed no earlier than 24–48 h after IVIg administration. Thus, it was surprising and unusual that vWF : Ag and vWF : Rco increased immediately (<8 h) after IVIg administration in our present case.

With regard to intracranial extramedullary involvement, a recently reported series of 50 patients included 14 patients with dural or extradural involvement at diagnosis [2]. Of these patients, 78% exhibited spinal osteolytic abnormalities, which were not observed in our present patient. Of the 50 patients, 10 were able to benefit from an autotransplant, including 7 who exhibited complete remission/very good partial response. Multivariate analysis in that study revealed that survival was associated with B2 microglobulin levels and complete response or very good partial response. The median survival was 25 months among patients with plasmacytoma exhibiting dural or extradural involvement and was 46 months among MM patients without CNS involvement.

In conclusion, the survival of patients with multiple myeloma and extradural disease remains lower than that of other MM patients. However, new therapeutic options and reinforcement with autotransplant have yielded encouraging results. Our currently described patient presented with a serious initial disease associated with acquired VWS, but highly effective treatment led to regression of the symptoms without sequelae.
